# Single-step hysteroscopic myomectomy for submucous leiomyoma

**DOI:** 10.4274/tjod.galenos.2020.64280

**Published:** 2020-07-29

**Authors:** Müge Keskin, Didem Çakmak, Aslı Yarcı Gürsoy, Aslıhan Alhan, Recai Pabuçcu, Gamze Sinem Çağlar

**Affiliations:** 1 Ufuk University Faculty of Medicine, Department of Obstetrics and Gynecology, Ankara, Turkey; 2 Ufuk University Faculty of Medicine, Department of Biostatistics, Ankara, Turkey

**Keywords:** Hysteroscopy, myomectomy, leiomyoma

## Abstract

**Objective:**

Leiomyomas are most commonly observed benign tumors in the female genital tract. Depending on the size, number, and location, the complete resection of Type 0, 1, and 2 leiomyomas by hysteroscopy can be completed in a single-step or multi-step procedure. The purpose of this study is to document the cases of hysteroscopic myomectomy performed via the resectoscopic technique in the gynecology department of a university hospital. Moreover, we assessed the applicability of single- or multi-step hysteroscopic myomectomy with respect to the diameter of the leiomyoma.

**Materials and Methods:**

We retrospectively reviewed the records of hysteroscopic myomectomy performed between 2012 and 2018. According to the diameter of the submucous leiomyomas, we divided 46 patients into 2 groups. Group 1 (n=25) consisted of patients with submucous leiomyomas <3 cm, whereas patients in group 2 (n=21) had submucous leiomyomas ≥3 cm in diameter. We recorded the number of removed leiomyomas and completed hysteroscopy sessions.

**Results:**

Myomectomy was completed by single-step hysteroscopy in all the patients of group 1, whereas eight patients in group 2 needed multiple sessions of hysteroscopy. None of the patients in group 1 had fluid overload; however, two patients in group 2 had mild asymptomatic hyponatremia.

**Conclusion:**

The success of hysteroscopic myomectomy depends on the diameter, localization, and number of the leiomyomas. This study revealed that Type 0, 1, and 2 leiomyomas of less than 3 cm can be resected by single-step hysteroscopy. For larger leiomyomas, the possibility of need for further sessions should be shared with the patients.

**PRECIS:** We have retrospectively evaluated the applicability of single- or multi-step hysteroscopic myomectomy regarding the diameter of the leiomyoma.

## Introduction

Leiomyomas are most commonly observed benign tumors in the female genital tract^(1)^, and they are observed in 70%-80% of women in reproductive age^(2)^. A total of eight types of leiomyomas are listed in the International Federation of Gynecology and Obstetrics (FIGO) classification system. According to the FIGO classification system, submucous leiomyomas are described in three groups. Type 0 refers to the completely intracavitary leiomyomas, Type 1 refers to the leiomyomas with the largest diameter in the uterine cavity, and Type 2 refers to the leiomyomas with the largest diameter in the myometrium^(3,4)^. Type 0, 1, and 2 leiomyomas make up about 5.5%-16.6% of all the uterine leiomyomas^(5)^ and are often associated with abnormal uterine bleeding, heavy menstrual bleeding, pelvic pain, dysmenorrhea, and infertility^(6)^. Hysteroscopic resection is the first-line treatment for intracavitary leiomyomas^(7)^.

Neuwirth and Amin^(8)^ first performed hysteroscopic myomectomy through the transcervical approach, and this procedure has become the gold standard treatment of choice^(8,9)^ for Type 0, 1, and 2 leiomyomas. Nevertheless, factors such as desire for future fertility; size, number, and location of the submucous leiomyomas; relationship of the deepest aspect of the myoma to the uterine serosa (in Type 2 leiomyomas); training, experience, surgical expertise of the surgeon, and availability of appropriate equipment affect the route of surgical intervention^(10)^. Experienced surgeons can remove leiomyomas of up to 4-5 cm in diameter under hysteroscopic direction. Depending on the above factors, the complete resection of Type 0, 1, and 2 leiomyomas by hysteroscopy can be completed in a single-step or multi-step procedure.

The hysteroscopic removal of the submucous leiomyomas might be carried out by different techniques such as resectoscopic excision by slicing, cutting of the base of the myoma and its extraction, ablation by Neodymium-doped Yttrium Aluminum Garnet (Nd: YAG) laser, and vaporization or morcellation by intrauterine morcellator^(11)^. The purpose of this study is to document the cases of hysteroscopic myomectomy performed via resectoscopic technique in the gynecology department of a university hospital. Moreover, we also assessed the applicability of single- or multi-step hysteroscopic myomectomy with respect to the diameter of the leiomyoma.

## Materials and Methods

Since the study consisted of retrospective analysis of the data, we did not have ethics committee approval.

We retrospectively reviewed the records regarding hysteroscopic myomectomy performed between 2012 and 2018 in a university gynecology department. According to the records, two expert surgeons having the same skill level and educational background performed the surgical procedures. We also analyzed the demographic variables and details regarding the hysteroscopic treatment for 46 patients. According to the diameter of the submucous leiomyomas, we divided the patients into two groups. Group 1 (n=25) consisted of patients who had submucous leiomyomas <3 cm in diameter, whereas patients in group 2 (n=21) had submucous leiomyomas ≥3 cm in diameter. Importantly, we recorded the data regarding the number of removed leiomyomas, number of completed hysteroscopic sessions, menopausal status, infertility status, and histopathological examination results of the specimens.

The patients were evaluated preoperatively by ultrasonography for the number, diameter, and the localization of the leiomyomas. Hysteroscopic myomectomy was performed under general anesthesia while using the monopolar loop resectoscope. Glycine 1.5% was used as the distension medium, and fluid deficit was observed throughout the operation in all the cases.

### Statistical Analysis

We performed the statistical analysis by using IBM® SPSS® Statistics for Windows version 22.0. We also performed Shapiro-Wilk tests for all the measurements to test for normality. Additionally, we compared the different groups by using the Mann-Whitney U test wherever it was appropriate. A p-value of less than 0.05 was considered statistically significant for this study.

## Results

In total, 46 patients underwent hysteroscopic myomectomy for Type 0, 1, and 2 leiomyomas. In total, 25 patients had submucous leiomyomas <3 in diameter (group 1), whereas 21 patients had submucous leiomyomas ≥3 cm in diameter (group 2). Table presents the demographic variables as well as operative and histopathological findings of the participants. Myomectomy was completed as single-step hysteroscopy in all patients of group 1, whereas 8 patients in group 2 needed multiple sessions of hysteroscopy (ranged between one and four) for complete excision. None of the patients in group 1 had fluid overload, whereas two patients in group 2 had mild asymptomatic hyponatremia.

## Discussion

Hysteroscopy is accepted as the gold standard treatment of choice for Type 0, 1, and 2 leiomyomas^(7)^. The success of the treatment mainly depends on the diameter, localization, and number of the leiomyomas but also on the experience of the surgeon and the available equipment. This study revealed that Type 0, 1, and 2 leiomyomas of less than 3 cm can be resected by single-step hysteroscopy by using monopolar energy modalities. For larger myomas and multiple myoma cases, more than one session might be needed for the complete resection to avoid fluid overload and other related complications when monopolar modalities are used. Single-step intervention was fully curative for only 61.9% of the cases with Type 0, 1, and 2 leiomyomas with size ≥3 cm.

In the latest French guidelines, the complete hysteroscopic resection of intracavitary leiomyomas was recommended as a first-line treatment for symptomatic Type 0, 1 (grade B), and 2 (grade C) leiomyomas up to 4 cm (grade C). But it was also stated that complete hysteroscopic resection was possible for fibroids of 4-6 cm. A two-stage resection was recommended in the incomplete first resection^(7)^. The diameter of the lesion appears to be the most important factor correlated with the need for multiple interventions due to the association between the diameter and the volume increments. For example, a myoma of 3x3x3 cm would have a volume of 13.5 cm^3^, whereas a myoma of 4x4x4 cm would have a volume of 32 cm^3^, which is almost twice as large than the previous one. Therefore, it was also previously stated that myoma diameters exceeding 3-5 cm were associated with the incomplete resection of leiomyomas and a further requirement for more than one hysteroscopy sessions^(12)^. A retrospective study including 1,244 patients who underwent hysteroscopic myomectomy using either cold loop technique or resectoscopic excision by slicing with a monopolar electric loop also revealed that the type and size of the myoma were significant for the discrimination between single-step and multi-step procedure. The authors also pointed out that leiomyomas with a diameter more than 3 cm were more often associated with multiple-step hysteroscopy^(13)^. It has already been revealed that submucous leiomyomas with a diameter more than 3-5 cm are better removed in the multiple sessions of hysteroscopy^(7)^. Our findings complies with the previous studies. Nevertheless, larger studies may clarify the exact cut-off values.

The limitation of hysteroscopic myomectomy is the risk of fluid overload. Fluid overload is more common in the cases of monopolar instrument employment because of non-conductive distending media usage^(10)^. In our study, all the cases were operated by monopolar energy, which necessitates the use of hypo-osmolar distention media. The utilization of bipolar instrument is associated with less fluid overload because electrolyte-containing solutions are less likely to cause undesired changes in the serum sodium levels and osmolality. Although the use of bipolar energy modalities decreases the risk of fluid overload, it is not available at our clinic. There were only two cases and both of them had asymptomatic hyponatremia. They were in group 2 and simply treated with loop diuretics. But it should be kept in mind that fluid overload might end up with even life-threatening serious complications.

### Study Limitations

The limitations of this study are a small sample size and a retrospective analysis. However, the results comply with the further studies, which support multiple-step intervention risk for Type 0, 1, and 2 leiomyomas bigger than 3 cm in diameter.

## Conclusion

The hysteroscopic resection of uterine leiomyoma is a well-tolerated, safe, and effective procedure. The sufficient preoperative evaluation and diagnosis is important for the selection of patient. Adequate instrumentation, careful fluid evaluation, and meticulous orientation of the anatomy will reduce complications. Each patient should be evaluated on an individual basis for the feasibility of the hysteroscopic approach, and the possibility of need for further sessions for the cases with submucous leiomyomas ≥3 cm in diameter should be shared with the patients before the procedure.

## Figures and Tables

**Table 1:**
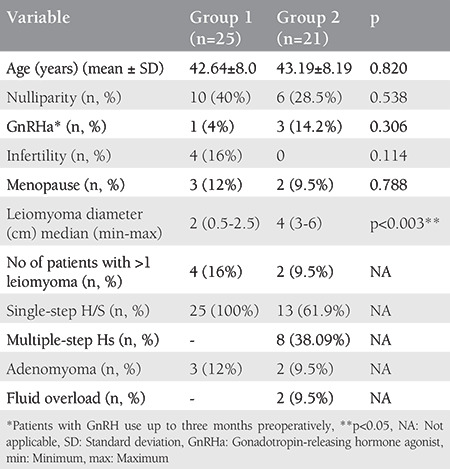
Characteristics and hysteroscopic findings of the patients
